# Paraneoplastic Syndromes in Neuroendocrine Prostate Cancer: A Systematic Review

**DOI:** 10.3390/curroncol31030123

**Published:** 2024-03-21

**Authors:** Mohammad Abufaraj, Raghad Ramadan, Amro Alkhatib

**Affiliations:** 1 Division of Urology, Department of Special Surgery, The University of Jordan, Amman 11942, Jordan; 2 School of Medicine, The University of Jordan, Amman 11942, Jordan

**Keywords:** prostate cancer, paraneoplastic, neuroendocrine tumor

## Abstract

Neuroendocrine prostate cancer (NEPC) is a rare subtype of prostate cancer (PCa) that usually results in poor clinical outcomes and may be accompanied by paraneoplastic syndromes (PNS). NEPC is becoming more frequent. It can initially manifest as PNS, complicating diagnosis. Therefore, we reviewed the literature on the different PNS associated with NEPC. We systematically reviewed English-language articles from January 2017 to September 2023, identifying 17 studies meeting PRISMA guidelines for NEPC and associated PNS. A total of 17 articles were included in the review. Among these, Cushing’s Syndrome (CS) due to ectopic Adrenocorticotropic hormone (ACTH) secretion was the most commonly reported PNS. Other PNS included syndrome of inappropriate Anti-Diuretic Hormone secretion (SIADH), Anti-Hu-mediated chronic intestinal pseudo-obstruction (CIPO), limbic encephalitis, Evans Syndrome, hypercalcemia, dermatomyositis, and polycythemia. Many patients had a history of prostate adenocarcinoma treated with androgen deprivation therapy (ADT) before neuroendocrine features developed. The mean age was 65.5 years, with a maximum survival of 9 months post-diagnosis. NEPC is becoming an increasingly more common subtype of PCa that can result in various PNS. This makes the diagnosis and treatment of NEPC challenging. Further research is crucial to understanding these syndromes and developing standardized, targeted treatments to improve patient survival.

## 1. Introduction

NEPC is an aggressive subtype of PCa that can arise de novo or develop from adenocarcinoma treated with ADT [1,2]. It is characterized by resistance to hormonal therapies, an aggressive clinical course, and an overall dismal prognosis [3]. Ineffective treatment regimens and delayed diagnosis are contributing factors to the poor prognosis of NEPC [1].

Approximately 1% of primary PCa exhibit neuroendocrine features and it has been estimated that at least 25% of patients with advanced PCa may experience neuroendocrine subtype development [4,5]. The classification system of NEPC includes the following six morphologic subtypes: 1—Prostate adenocarcinoma with neuroendocrine differentiation, 2—Carcinoid tumor, 3—Small cell carcinoma, 4—Large cell neuroendocrine carcinoma, 5—Adenocarcinoma with Paneth cell neuroendocrine differentiation, and 6—Mixed neuroendocrine carcinoma–acinar adenocarcinoma [3].

Neuroendocrine tumors are distinguished by their ability to produce and secrete biologically active compounds distinctive to the cell of origin, which can result in paraneoplastic syndromes (PNS) [6]. PNS are described as a group of symptoms and clinical signs that are not linked either to the tumor’s local effects or distant spread [7]. PCa is the second most common urological tumor associated with PNS, after renal cell carcinoma [7]. NEPC can cause a variety of paraneoplastic manifestations, including endocrine PNS such as CS and SIADH, in addition to dermatomyositis, polycythemia, and CIPO [7,8,9].

Recognizing NEPC and associated PNS is crucial because the symptoms and signs of PNS may be the first presentation of a previously undiagnosed neoplasm, and this can mislead the identification of the origin of a primary tumor [7]. In addition, NEPC has become an increasingly more common subtype of PCa [3,7,10]. In this article, we systematically review the literature on PNS associated with NEPC, their presentation, the investigations carried out, modes of treatment offered, and outcomes. This assessment aims to identify gaps in the literature, thereby facilitating the identification of the areas warranting further exploration and paving the way for future research endeavors, seeking a deeper understanding of PNS associated with NEPC.

## 2. Materials and Methods

### 2.1. Literature Search

We followed the Preferred Reporting Items for Systematic Review and Meta-analyses (PRISMA) guidelines, consisting of a checklist including 27 items (Supplementary Material S1), in conducting this systematic review [11]. This systematic review was pre-registered in the International Prospective Register of Systematic Reviews (PROSPERO) database (CRD42023455770).

To ensure the originality of our review within the current literature, we conducted a literature search of articles published from January 2017 to September 2023 in the PubMed database by combining the following PICO (patient population, intervention, comparison, outcome) terms for population: “neuroendocrine prostate cancer”, “carcinoid tumor of the prostate”, “small cell prostate cancer”, “large cell neuroendocrine carcinoma of the prostate”, “Prostate adenocarcinoma”. Using an AND operator we combined those terms with the following terms for outcomes of paraneoplastic syndromes: “Paraneoplastic syndrome”, “Paraneoplastic syndromes”, “Paraneoplastic endocrine syndromes”, “Paraneoplastic endocrine syndrome”, “Paraneoplastic polyneuropathy”, “Paraneoplastic polyneuropathies”, “Paraneoplastic cerebellar degeneration”, “Ectopic ACTH syndrome”, “Hypercalcemia”, “Parathyroid hormone-related peptide”, “Stauffer syndrome”, “Guillain-Barré syndrome”, “Paraneoplastic hypercalcemia”, “Paraneoplastic neurological syndromes”, “Paraneoplastic neurological syndrome”, “Paraneoplastic manifestations”, “Paraneoplastic manifestation”, “Paraneoplastic limbic encephalitis”, “Limbic encephalitis”, “Paraneoplastic endocrinopathy”, “Paraneoplastic endocrinopathies”, “Ocular paraneoplastic syndromes”, “Ocular paraneoplastic syndrome”, “SIADH”, “Syndrome of inappropriate ADH secretion”, “Syndrome of inappropriate anti diuretic hormone secretion”, “Cushing’s Syndrome”, “Paraneoplastic cushing syndrome”, “Hematological disorders”, “DIC”, “Neurological syndromes”, “LEMS”, “Lambert-Eaton myasthenic syndrome”, “peripheral neuropathy”, “cerebellar degeneration”, “Stauffer’s syndrome”.

In addition, reference sections of the included articles were screened, and relevant articles were incorporated into the review. The search aimed to address our primary objective of compiling and summarizing the existing medical literature concerning PNS that occur in association with NEPC, their variable presentations, management options, and the survival of the patients.

### 2.2. Inclusion Criteria

Articles were considered eligible for inclusion if they (1) referred to patients diagnosed with an NEPC, or a prostate adenocarcinoma with neuroendocrine differentiation proven by histology of the original tumor or one of its metastatic lesions, or through 68Gallium-DOTATATE PET–CT scan, or patients with a history of prostate malignancy who were found to have a neuroendocrine component on a metastatic lesion histologically, believed by the authors to be caused by the prostate malignancy; (2) referred to patients who developed a PNS, defined as “rare clinical syndromes due to the systemic effects of tumors; they are unrelated to tumor size, invasiveness or metastases”, where there was no explanation for the presentation other than a PNS attributed to the NEPC malignancy [12]. (3) Articles published in the English language.

### 2.3. Study Selection and Data Extraction

Study selection was carried out by RR and AA, who independently evaluated the search results against our inclusion criteria. Any discrepancies were resolved through discussion with the senior author, MA.

We extracted the following variables of interest from the included studies: first author’s name, patient’s age, presentation, the PNS, treatment of the PNS, prior ADT, Prostate Specific Antigen (PSA) levels, and histology and mortality of the patients when reported. Data extraction was carried out by RR and AA independently using Mendeley and Excel and then checked by MA.

Any discrepancy or disagreement was solved by discussion. In cases where agreement could not be reached, a resolution was determined based on the majority opinion following consultation with the senior author MA. Data were extracted into an Excel sheet.

### 2.4. Quality Assessment

The quality of the articles was assessed by two authors, RR and AA, independently using Joanna Briggs Institute (JBI) critical appraisal checklist for case reports [13]. This tool consists of 8 questions as follows: “(Q.1) Were patients’ demographic characteristics clearly described?, (Q.2) Was the patient’s history clearly described and presented as a timeline?, (Q.3) Was the current clinical condition of the patient on presentation clearly described?, (Q.4) Were diagnostic tests or assessment methods and the results clearly described?, (Q.5) Was the intervention(s) or treatment procedure(s) clearly described?, (Q.6) Was the post-intervention clinical condition clearly described?, (Q.7) Were adverse events (harms) or unanticipated events identified and described?, (Q.8) Does the case report provide takeaway lessons?” Each question received one of four answers—yes (score: 1), unclear (score: 0.5), no (score: 0), or not applicable—and each article had a score out of 8. The articles were classified into three categories as follows: low quality (score: ≤4.5), moderate quality (score: 5–6.5), and high quality (score: 7–8). Any disagreement between the authors was resolved by consensus and discussion with the senior author MA. Articles of high and moderate quality were included. Fourteen articles (82.3%) were of high quality and three articles (17.6%) were of moderate quality (Table 1).

## 3. Results

**Figure 1:**
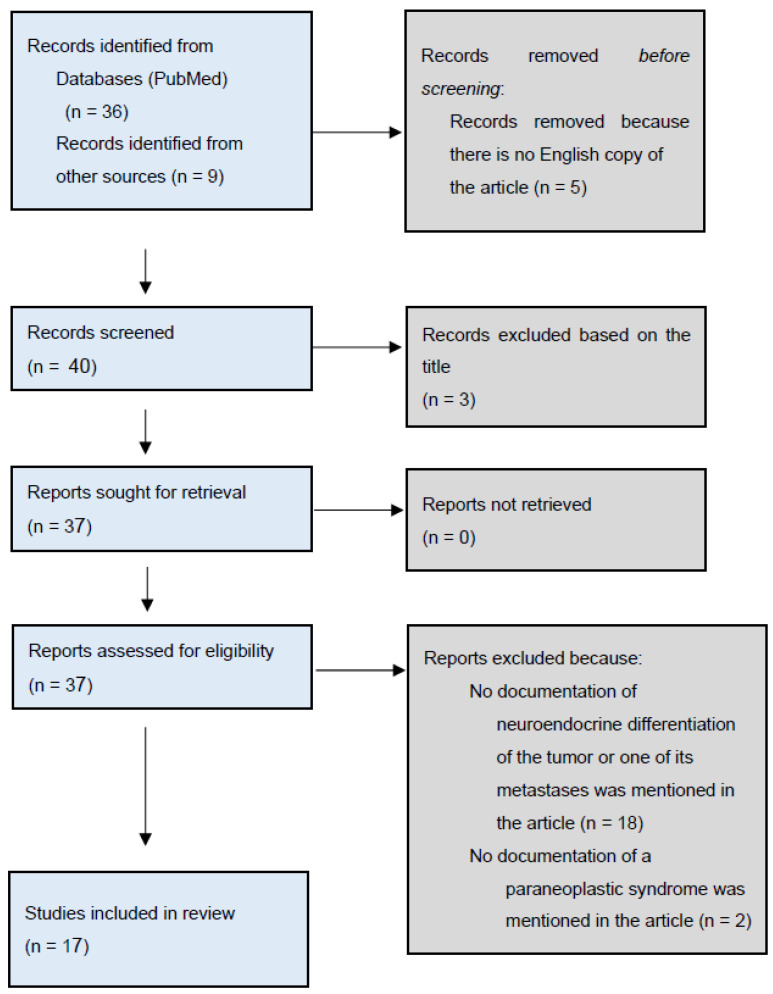
Flow diagram of the search results.

Our search strategy yielded 36 articles in addition to 9 articles found through screening the references of the included studies and other sources such as Google Scholar. Five articles were excluded because they were not available in English. After screening the titles of the remaining 40, another 3 articles were excluded. The remaining 37 articles were retrieved and reviewed in-depth, of which 20 articles were removed because they did not meet our inclusion criteria. Consequently, 17 articles were included, all of which turned out to be case reports (Figure 1). The excluded articles and justifications for their exclusion can be found in Supplementary Material S2.

Table 2 summarizes the 17 case reports included in this systemic review. Eight different PNS developing from NEPC were identified. These included CS due to ectopic ACTH secretion, hypercalcemia, Evans Syndrome (the presence of two or more immune cytopenias) [27,28], limbic encephalitis, CIPO, SIADH, dermatomyositis, and polycythemia. The mean age of patients was 65.5.

CS was the most common PNS, and was found in 11 out of 17 cases. There were several presentations, but hypokalemia, resistant hypertension, and fluid overload were documented frequently. Furthermore, 7 out of 11 patients with paraneoplastic CS had a previous history of prostate adenocarcinoma, for which they were treated with ADT before developing neuroendocrine differentiation and CS [10,16,17,18,19,20,21]. Small cell carcinoma was the predominant histological subtype, with one case having a large cell NEPC [14,15,19,20,21,22,23]. In addition, Prostate Specific Antigen (PSA) levels were high in patients who reported a previous history of prostate adenocarcinoma [10,16,18,20,21] but were normal in patients who developed prostate neuroendocrine cancer de novo [14,15,23]. The management of paraneoplastic CS was mostly based on Ketoconazole, Metyrapone, Spironolactone, and Potassium supplementation, along with treating hyperglycemia and hypertension. Surgical management represented by urgent bilateral adrenalectomy was needed in one case to control severe hypercortisolism before starting medical treatment [15]. Eventually, most patients passed away within 9 months of their diagnosis and documented causes of death included infections and multiple organ failure.

Evans Syndrome [27], Limbic encephalitis [26], Hypercalcemia [19], CIPO [8], SIADH [24,25], dermatomyositis, and polycythemia [9] were the other seven PNS identified in our review. As with CS, the histological subtype in all of these syndromes was small-cell neuroendocrine cancer, except for dermatomyositis and polycythemia where it was large-cell carcinoma [8,9,25,26,27]. A previous history of prostate adenocarcinoma treated with ADT was identified in three out of the six cases, these patients passed away at a maximum of five months from their presentation.

## 4. Discussion

PCa is the second most common urological malignancy, known for its ability to cause PNS after renal cell carcinoma, especially in the advanced stage [7,29]. Neuroendocrine differentiation is a frequent finding in the histology of prostate malignancies that have paraneoplastic manifestations [29]. A variety of endocrine, neurologic, rheumatologic, and hematologic syndromes were identified in association with neuroendocrine PCa in our review.

Paraneoplastic CS due to ACTH secretion constitutes 10–20% of CS cases [14,22]. PCa, though rare, forms an important cause of rapidly progressive CS [14].

As demonstrated in Table 2, we found that CS is the most frequently reported PNS associated with NEPC. Patients presented in a variety of ways, including fluid overload, hypokalemia, resistant hypertension, metabolic alkalosis, easy bruising, fatigue, and generalized weakness [10,14,15,16,17,18,20,21,22,23]. On physical examination, many of the reviewed cases [14,15,16,17,18,20,21] had proximal weakness and edema, although the classical features of CS are usually absent in malignancy [30].

Notably, most of the patients with ectopic ACTH secretion had a history of prostate adenocarcinoma treated with ADT, and the dominant histological subtype of prostate neuroendocrine cancer was small cell (Table 2). Around 40–50% of patients who had small cell NEPC were found to have a history of adenocarcinoma of the prostate and it is believed that these two tumors have a common origin [31]. Furthermore, ADT increasing the risk of neuroendocrine differentiation of a previous prostate adenocarcinoma is a well-documented phenomenon, although the true mechanisms behind this are not clear [32]. It has been proposed that certain stem cells differentiate into both adenocarcinoma and neuroendocrine cells; subsequently, ADT exerts its effect on hormone-responsive cells, thereby permitting neuroendocrine cells to progress into NEPC. Conversely, certain experiments indicate that NEPC undergoes transdifferentiation from prostatic adenocarcinoma, supported by the detection of prostate cancer-specific mutations (for example, ERG rearrangements) and gene amplifications (for example, AURKA and *MYCN*) in both subtypes. This evidence supports a shared etiology between these two malignancies [31]. Among patients with a previous history of prostate adenocarcinoma that underwent neuroendocrine differentiation and where the grade of the cancer was recorded, two of them had a high-grade prostate adenocarcinoma [14,17].

Prostate-specific antigen (PSA) values are usually normal in men with small cell cancer [31]. This was also the case in our review (as shown in Table 2), except for patients with a previous history of adenocarcinoma, who had elevated PSA levels [10,16,18,20,21]. The reviewed cases emphasize the concept that negative immunohistochemical staining for PSA or ACTH should not exclude an ectopic ACTH-secreting prostate neuroendocrine tumor as this subtype of PCa usually loses or partially expresses PSA, as it is reported that up to 30% of ACTH-secreting tumors stain negative for ACTH [10,17,19,20,21,27,33,34].

The diagnosis of CS requires laboratory and imaging studies considering the possibility of an ectopic source secreting ACTH. Pituitary Magnetic Resonance Imaging (MRI) is a fundamental part of the workup to exclude a pituitary mass. A thorough radiological review of the imaging is required, as Elston et al. reported a case where an enlarged prostate was missed for the first time on a Computed Tomography (CT) scan of a patient. However, a second careful review of the scan identified the mass, establishing the diagnosis of paraneoplastic CS [14].

Many drugs are available for the management of ectopic ACTH secretion, but no specific guidelines are stated. Ketoconazole and/or metyrapone are recommended and frequently used; mitotane is another medical option, but with a slower onset of action [35,36]. Etomidate can be administered in a parenteral route [35]. Other medications, including Mifepristone and Cabergoline, were also used [18,20].

Bilateral adrenalectomy is a lifesaving surgery that can be performed to control severe hypercortisolism [36]. Klomjit et al. reported a case where surgical management was needed before commencing medical management [15]. Following treatment, clinical and biochemical follow-up of the patient is crucial [36].

The prognosis of patients with paraneoplastic CS due to NEPC is poor (Table 2). Patients survived for no longer than 9 months from their diagnosis of PNS. This aligns with the findings outlined in the latest review on NEPC conducted by Elston et al., where the survival of patients who received both adrenal blockade and chemotherapy was 9 months, compared to 2 months for patients treated with only blocking the adrenals and less than 1 month for patients without treatment [14] Elston et al. documented sepsis as the predominant cause of mortality in their reported cases [14]. Consistent with their findings, our review identified infections and multiple organ failure as the primary causes leading to mortality. It is well established that patients with hypercortisolism are at increased risk of infections, and with these infections spreading and becoming more generalized, sepsis and multiple organ failure ensue [10,14,17,22,23,37].

Interestingly, one case exhibited concurrent paraneoplastic hypercalcemia and paraneoplastic CS. The patient manifested symptoms of constipation, anorexia, and imbalance. Laboratory findings indicated elevated serum calcium levels, normal parathyroid hormone (PTH), low 25-OH vitamin D, and elevated PTH-related peptide (PTHrP). The patient’s hypercalcemia resolved after the administration of Pamidronate [38]. Management should include addressing the underlying cause along with discontinuing medications contributing to hypercalcemia. The first line in the management of persistent hypercalcemia includes fluid repletion with normal saline. Other treatment options include Bisphosphonates, Calcitonin, Mithramycin, Gallium nitrate, and hemodialysis, particularly for patients with significant renal or cardiac disease [38].

Our study included PNS beyond CS. For instance, dermatomyositis is a rheumatologic condition that is commonly associated with malignancies. It is thought to be caused by antibodies directed against tumor antigens that cross-react with muscle antigens [39]. Moreover, the ectopic production of Erythropoietin (EPO) causing erythrocytosis is another PNS that is usually related to hepatic or renal cell cancer [40,41]. Papagoras et al. reported a case of large cell NEPC that presented with facial redness and swelling, in addition to complaints of fatigue, that was managed as an allergic case with steroids, but the patient’s symptoms came back a few weeks later. Investigations revealed a prostate mass along with polycythemia. The patient was then diagnosed with metastatic PCa with paraneoplastic dermatomyositis and polycythemia. Surprisingly, Erythropoietin levels were low normal [9]. This could be explained by the negative feedback mechanism of the abnormally secreted EPO from the tumor cells.

CIPO is a rare syndrome that results in chronic intestinal dysmotility and nutrient malabsorption [8,42]. This syndrome is associated with the presence of anti-Hu antibodies, which are hypothesized to be directed against an epitope that is present in the malignancy as well as the neurons of the enteric nervous system [43]. It is believed that these antibodies work directly through activating the nerves by nicotinic receptors, thus activating visceral afferents and causing gut dysmotility [44]. This syndrome is usually associated with small-cell lung cancer [8,42]. Cerra-Franco et al. reported a patient with small-cell PCa who was diagnosed with anti-Hu mediated paraneoplastic CIPO who presented with constipation and abdominal distention. It was presumed that his symptoms were caused by peritoneal carcinomatosis, but no mechanical cause was identified on the repeated abdominopelvic CT scan [8]. He was managed supportively with IV fluids and bowel rest. Eventually, a gastrostomy was placed, as there is no current effective treatment for CIPO [8,45]. The optimal way to deal with this syndrome is to treat the malignancy early on to prevent irreversible neuronal damage [46].

Metastatic prostate adenocarcinoma is a frequently recognized malignancy associated with the paraneoplastic SIADH [29]. It is usually a high-grade tumor with acquired neuroendocrine features [25,29]. As shown in Table 2, presentations of SIADH included mental confusion, fatigue, dizziness, and the presence of hyponatremia [24,25]. Both documented cases of SIADH exhibited a neuroendocrine component upon biopsy analysis [24,25]. These cases support the belief that the evolution of neuroendocrine characteristics in PCa increases tumor invasiveness and the risk of developing PNS [24,25]. Both patients were managed with oral tolvaptan, a vasopressin-2 receptor antagonist, which is found to improve serum sodium concentration, but with little data on its impact on the survival of patients with malignancy [47,48]. The mean survival reported in the literature for such cases is under one year [29]. As shown in Table 2, liver failure was the cause of death of one patient 5 months following his diagnosis with SIADH.

Limbic encephalitis is a rare paraneoplastic neurologic syndrome that develops as an autoimmune response against neural antigens [26]. Several antibodies were identified in association with this syndrome, including type B gamma-aminobutyric acid receptor (GABA-B) antibodies, which are found in 5% of autoimmune encephalitis cases and carry a better prognosis than other antibodies [26,49,50,51]. The vague presenting symptoms of this syndrome, in addition to the profile of patients who are usually elderly with advanced malignancy, make the diagnosis challenging [26]. In a reported case of a 66-year-old male, a diagnosis of prostate adenocarcinoma was made. The patient received ADT. After 13 years, he presented with generalized tonic–clonic seizures followed by postictal confusion. A diagnosis of limbic encephalitis was made with the biopsy showing small-cell prostate neuroendocrine cancer [26]. Management including treating the underlying tumor, as well as antiepileptics, IV steroids, immunotherapy, and plasmapheresis, may be necessary [26].

Evans syndrome is another rare paraneoplastic syndrome that usually occurs in the setting of hematological malignancies [27]. It is characterized by the presence of two or more immune cytopenias, commonly autoimmune hemolytic anemia (AHIA) and immune-mediated thrombocytopenia [27,28]. Solid tumors including prostate malignancies are uncommon causes of this syndrome [27,52]. Sidda et al. reported a case of prostate adenocarcinoma, which was treated with ADT. The patient presented 6 months following his diagnosis with fatigue and was found to have anemia and thrombocytopenia. A Coombs test was negative, demonstrating one of the diagnostic difficulties associated with this syndrome. The patient was treated with IV steroids and immunoglobulins. Then, he underwent a bone marrow biopsy that showed a transformation into small-cell neuroendocrine cancer [27]. Evans syndrome is usually difficult to treat and causes frequent relapses [53].

Upon our examination of the 17 cases included in our study, it was revealed that 8 cases of prostate adenocarcinoma had been diagnosed with a range of 6–156 months before the manifestation of PNS. In contrast, Elston et al. reported a range of 2–48 months [14]. This discrepancy may stem from our review encompassing cases beyond CS, which could potentially result in delayed presentations. For instance, we observed a case of limbic encephalitis that manifested 13 years after the diagnosis of adenocarcinoma.

This is the most updated systematic review of PNS occurring from NEPC. This study adhered to the guidelines instituted by the PRISMA. However, several limitations must be acknowledged. Primarily, the available body of literature on the PNS associated with NEPC is limited. Furthermore, all of these studies were case reports, which resemble low levels of evidence. This underscores the importance of conducting future research that employs more robust study designs to comprehensively understand PNS associated with NEPC. Lastly, our study only included articles published in English, thereby restricting the comprehensive inclusion of all relevant studies available on this subject in the literature.

## 5. Conclusions

NEPC is becoming a more common subtype of PCa with the potential to cause plenty of PNS such as CS, Evans Syndrome, limbic encephalitis, CIPO, SIADH, dermatomyositis, and polycythemia. The ambiguity of the presenting complaints, in addition to the rapid clinical deterioration course, make the diagnosis and treatment challenging. Thus, it is crucial to recognize these syndromes to promptly manage the patients and prevent further complications. We encourage practicing physicians to consider neuroendocrine differentiation and PNS in patients with a history of prostate adenocarcinoma treated with ADT, since most of the reported cases of neuroendocrine PCa in our review had a history of prostate adenocarcinoma treated with ADT and this is a frequently reported phenomenon in the literature. Further research is warranted to identify the true mechanisms behind the neuroendocrine differentiation of prostate tumors and to understand the exact pathophysiology of these syndromes. Consequently, more standardized and targeted treatments for PNS caused by prostate neuroendocrine cancer can be formulated, leading to improved overall patient survival.

## Figures and Tables

**Table 1:** Quality assessment of included studies.

Study	Question 1	Question 2	Question 3	Question 4	Question 5	Question 6	Question 7	Question 8	Score
Elston et al. (2017) [14]	Yes	Yes	Yes	Yes	Yes	Yes	Unclear	Yes	7.5/8
Klomjit et al. (2019) [15]	Yes	Yes	Yes	Yes	Yes	Yes	Unclear	Yes	7.5/8
Zeng et al. (2022) [16]	Yes	Yes	Yes	Yes	Yes	Yes	Unclear	Yes	7.5/8
Hassan et al. (2022) [17]	Yes	Yes	Yes	Yes	Yes	Yes	Unclear	Yes	7.5/8
Tan et al. (2023) [18]	Yes	Yes	Yes	Yes	Yes	Yes	Yes	Yes	8/8
Feffer et al. (2018) [19]	Yes	Yes	Yes	Yes	Yes	Yes	Yes	Yes	8/8
Soundarrajan et al. (2019) [20]	Yes	Yes	Yes	Yes	Yes	Yes	Yes	Yes	8/8
Murphy et al. (2019) [10]	Yes	Yes	No	Yes	Yes	Yes	Unclear	Yes	6.5/8
Schepers et al. (2020) [21]	Yes	Yes	Yes	Yes	Yes	Yes	Unclear	Yes	7.5/8
Fernandes et al. (2021) [22]	Yes	Yes	Yes	Yes	Yes	Yes	Unclear	Yes	7.5/8
Riaza Montes et al. (2021) [23]	Yes	Yes	Yes	Yes	Yes	No	Unclear	Yes	6.5/8
Papagoras et al. (2018) [9]	Yes	Yes	Yes	Yes	No	Unclear	Unclear	Yes	6/8
Cerra-Franco et al. (2019) [8]	Yes	Yes	Yes	Yes	Yes	Yes	Yes	Unclear	7.5/8
Peverelli et al. (2017) [24]	Yes	Yes	Yes	Yes	Yes	Yes	Unclear	Yes	7.5/8
Fiordoliva et al. (2019) [25]	Yes	Yes	Yes	Yes	Yes	Yes	Unclear	Yes	7.5/8
Karray et al. (2021) [26]	Yes	Yes	Yes	Yes	Yes	Yes	Yes	Yes	8/8
Sidda et al. (2022) [27]	Yes	Yes	Yes	Yes	Yes	Yes	Unclear	Yes	7.5/8

**Table 2:** Summary of published cases of PNS associated with NEPC.

Author	Patient Age	Presentation	Paraneoplastic Syndrome	Treatment of the Paraneoplastic Syndrome	Prior Androgen Deprivation Therapy	PSA Levels at the Time of Presentation of Paraneoplastic Syndrome	Histology (Site of Sample)	Mortality
Elston et al. (2017) [14]	71	Hypokalemia, edema and new onset hypertension	Cushing’s Syndrome	Ketoconazole, Metyrapone, Spironolactone, Potassium supplementation	No	Normal	De novo high-grade (Gleason 5 + 5) acinar adenocarcinoma and a small-cell NE PC. (prostate)	After 9 months
Klomjit et al. (2019) [15]	69	Progressive bilateral lower leg edema, easy bruising, fatigue, generalized weakness and new onset severe resistant hypertension	Cushing’s Syndrome	Urgent bilateral adrenalectomy to control severe hypercortisolism, Spironolactone and antihypertensives	No	Normal	De novo Small cell NE PC with a small percentage of prostatic adenocarcinoma (prostate)	After 1 month from the progression of cancer
Zeng et al. (2022) [16]	61	Recurrent hypokalemia, lower limb weakness, and edema	Cushing’s Syndrome	Ketoconazole, Potassium supplementation, Spironolactone, antihypertensives, Metformin and Sitagliptin and Octreotide	Yes	Elevated	Acinar adenocarcinoma of the prostate with NE differentiation (prostate)	After 3 months.
Hassan et al. (2022) [17]	early 60′s	Abdominal distention, facial and bilateral upper limb and lower limb edema	Cushing’s Syndrome	Spironolactone and Mitotane	Yes	-	High-grade prostate adenocarcinoma with NE differentiation (Liver mets)	Due to multiple infections.
Tan et al. (2023) [18]	64	Recurrent fluid overload, severe hypokalemia with metabolic alkalosis and loss of glycemic control.	Cushing’s Syndrome	Ketoconazole, Cabergoline, fluid restriction, Spironolactone, Furosemide, Potassium replacement and Apixaban (replaced then with warfarin), Metformin and Insulin	Yes	Elevated	Prostate adenocarcinoma with NE differentiation. * (prostate)	Within 2 weeks from progression of cancer
Feffer et al. (2018) [19]	56	Clogged nephrostomy tubes	Cushing’s syndrome and PTHrP-mediated hypercalcemia	Pamidronate, Ketoconazole, Spironolactone, Insulin, and anti-hypertensives	Yes	-	High-grade small cell carcinoma (Liver mets)	-
Soundarrajan et al. (2019) [20]	73	Severe weakness, hyperglycemia, and hypokalemia	Cushing’s Syndrome	Ketoconazole, Metyrapone, Mifepristone, Spironolactone, Amlodipine, Insulin, and stress doses of steroids	Yes	Elevated	Small cell carcinoma (liver mets) and high-grade NE cancer (pleural fluid).	-
Murphy et al. (2019) [10]	63	Polydipsia, polyuria, and lower limb swelling	Cushing’s Syndrome	Ketoconazole	Yes	Elevated	NE PC (left inguinal lymph node)	After 1 month.
Schepers et al. (2020) [21]	75	Peripheral edema, metabolic alkalosis, hypokalemia, and hypertension	Cushing’s Syndrome	Ketoconazole, potassium chloride, and Spironolactone	Yes	Elevated	Large cell NE PC (prostate)	After several weeks.
Fernandes et al. (2021) [22]	56	Pelvic pain, rectal tenesmus, and fatigue	Cushing’s Syndrome	Metyrapone, Potassium supplementation, and anti-hypertensive medication	No	Elevated	De novo Small cell NE PC (prostate)	Within days from nosocomial UTI and multiple organ dysfunction
Riaza Montes et al. (2021) [23]	65	Acute urinary retention, arterial hypertension, and edema in lower extremities.	Cushing’s Syndrome	Ketoconazole, Etomidate, and potassium chloride	No	Normal	De novo Undifferentiated small cell PC (prostate)	After 21 days from septic shock with multiple organ failure and cardiac arrest
Papagoras et al. (2018) [9]	69	Facial erythema and symptoms of fatigue	Dermatomyositis and polycthemia	-	No	Elevated	De novo Large cell NE PC (prostate)	After 4 months.
Cerra-Franco et al. (2019) [8]	75	Worsening abdominal distention, constipation, voiding difficulty, and loss of appetite	Anti-Hu-Mediated Paraneoplastic Chronic Intestinal Pseudo-Obstruction	IV fluids, bowel rest, TPN, and gastrostomy	No	-	De novo Small cell NE PC (prostate)	After 6 weeks.
Peverelli et al. (2017) [24]	71	Fatigue, dizziness, and hyponatremia	SIADH	IV electrolytes supplementation, Tolvaptan	No	Normal	Prostate adenocarcinoma (Gleason 3 + 3) transformed into small cell PC (prostate)	-
Fiordoliva et al. (2019) [25]	60	Constipation and mild mental confusion	SIADH	Tolvaptan	Yes	Elevated	Acinar adenocarcinoma with NE differentiation (prostate). Small cell NE carcinoma. (Liver mets)	After 5 months due to liver failure
Karray et al. (2021) [26]	66	Generalized tonic–clonic seizures, followed by postictal confusion.	Limbic Encephalitis	Antiepileptics, methylprednisolone, Immunoglobulins, cyclophosphamide, and rituximab	Yes	Normal	Small cell NE PC (prostate)	-
Sidda et al. (2022) [27]	63	Fatigue, hemolytic anemia, and thrombocytopenia	Evans Syndrome	IV methylprednisone and immunoglobulin	Yes	-	Prostatic adenocarcinoma (Gleason 5 + 5) (Prostate). Metastatic PC with transformation to small cell NE carcinoma (Bone marrow)	Within a few days

NE, Neuroendocrine; PC, Prostate Cancer; SIADH, Syndrome of Inappropriate ADH secretion; IV, intravenous; TPN, Total parenteral nutrition. *: 68-Gallium-DOTATATE PET–CT scan confirmed neuroendocrine differentiation of prostate cancer with patchy DOTATATE uptake in the prostate bed. Histological diagnosis was not conducted in this patient because it would not affect the management plan.

## Supplementary Materials

The following are available online at https://www.mdpi.com/article/10.3390/curroncol31030123/s1: Supplementary Material S1: PRISMA 2020 Checklist [54]; Supplementary Material S2: List of Excluded Studies [55,56,57,58,59,60,61,62,63,64,65,66,67,68,69,70,71,72,73,74,75,76,77,78,79,80,81,82].
