# Supplementary material for: Paraneoplastic Syndromes in Neuroendocrine Prostate Cancer: A Systematic Review
Source: Curr Oncol. 2024 Mar 21;31(3):1618–32. doi: 10.3390/curroncol31030123 (PMC10969281; doi:10.3390/curroncol31030123)
Supplement: Supplementary file 1 [file curroncol-31-00123-s001.zip › curroncol-2881751-supplementary.pdf]

Supplementary Materials

Spplementary Material S1

PRISMA 2020 Checklist

Section and Topic	Item #	Checklist item	Location where item is reported
**TITLE**			
Title	1	Identify the report as a systematic review.	Page 1 Line 2
**ABSTRACT**			
Abstract	2	See the PRISMA 2020 for Abstracts checklist.	Page 1 Lines 8-25
**INTRODUCTION**			
Rationale	3	Describe the rationale for the review in the context of existing knowledge.	Pages 1-2 Lines 32-56
Objectives	4	Provide an explicit statement of the objective(s) or question(s) the review addresses.	Page 2 Lines 56-61
**METHODS**			
Eligibility criteria	5	Specify the inclusion and exclusion criteria for the review and how studies were grouped for the syntheses.	Page 3 Lines 94-104
Information sources	6	Specify all databases, registers, websites, organisations, reference lists and other sources searched or consulted to identify studies. Specify the date when each source was last searched or consulted.	Pages 2-3 Lines 63-70 89-93
Search strategy	7	Present the full search strategies for all databases, registers and websites, including any filters and limits used.	Pages 2-3 Lines

Section and Topic	Item #	Checklist item	Location where item is reported
			72-88
Selection process	8	Specify the methods used to decide whether a study met the inclusion criteria of the review, including how many reviewers screened each record and each report retrieved, whether they worked independently, and if applicable, details of automation tools used in the process.	Page 3 Lines 105-108
Data collection process	9	Specify the methods used to collect data from reports, including how many reviewers collected data from each report, whether they worked independently, any processes for obtaining or confirming data from study investigators, and if applicable, details of automation tools used in the process.	Page 3 Lines 109-116
Data items	10a	List and define all outcomes for which data were sought. Specify whether all results that were compatible with each outcome domain in each study were sought (e.g. for all measures, time points, analyses), and if not, the methods used to decide which results to collect.	Page 3 Lines 94-104
	10b	List and define all other variables for which data were sought (e.g. participant and intervention characteristics, funding sources). Describe any assumptions made about any missing or unclear information.	Page 3 Lines 109-113
Study risk of bias assessment	11	Specify the methods used to assess risk of bias in the included studies, including details of the tool(s) used, how many reviewers assessed each study and whether they worked independently, and if applicable, details of automation tools used in the process.	Page 3 Lines 117-133
Effect measures	12	Specify for each outcome the effect measure(s) (e.g. risk ratio, mean difference) used in the synthesis or presentation of results.	Not Applicable
Synthesis methods	13a	Describe the processes used to decide which studies were eligible for each synthesis (e.g. tabulating the study intervention characteristics and comparing against the planned groups for each synthesis (item #5)).	Page 3 Lines 94-104
	13b	Describe any methods required to prepare the data for presentation or synthesis, such as handling of missing summary statistics, or data conversions.	Not Applicable
	13c	Describe any methods used to tabulate or visually display results of individual studies and syntheses.	Page 3 Line 116
	13d	Describe any methods used to synthesize results and provide a rationale for the choice(s). If meta-analysis was performed, describe the model(s), method(s) to identify the presence and extent of statistical heterogeneity, and software package(s) used.	Not Applicable

Section and Topic	Item #	Checklist item	Location where item is reported
	13e	Describe any methods used to explore possible causes of heterogeneity among study results (e.g. subgroup analysis, meta-regression).	Not Applicable
	13f	Describe any sensitivity analyses conducted to assess robustness of the synthesized results.	Not Applicable
Reporting bias assessment	14	Describe any methods used to assess risk of bias due to missing results in a synthesis (arising from reporting biases).	Page 3 Lines 117-133
Certainty assessment	15	Describe any methods used to assess certainty (or confidence) in the body of evidence for an outcome.	Not Applicable
**RESULTS**			
Study selection	16a	Describe the results of the search and selection process, from the number of records identified in the search to the number of studies included in the review, ideally using a flow diagram.	Page 5 Lines 138-177
	16b	Cite studies that might appear to meet the inclusion criteria, but which were excluded, and explain why they were excluded.	Page 3 Lines 144-145, Supplementary material 2
Study characteristics	17	Cite each included study and present its characteristics.	Pages 7-11
Risk of bias in studies	18	Present assessments of risk of bias for each included study.	Page 4
Results of individual studies	19	For all outcomes, present, for each study: (a) summary statistics for each group (where appropriate) and (b) an effect estimate and its precision (e.g. confidence/credible interval), ideally using structured tables or plots.	Pages 7-11
Results of	20a	For each synthesis, briefly summarise the characteristics and risk of bias among contributing studies.	Page 6 Lines

Section and Topic	Item #	Checklist item	Location where item is reported
syntheses			180-207
	20b	Present results of all statistical syntheses conducted. If meta-analysis was done, present for each the summary estimate and its precision (e.g. confidence/credible interval) and measures of statistical heterogeneity. If comparing groups, describe the direction of the effect.	Not Applicable
	20c	Present results of all investigations of possible causes of heterogeneity among study results.	Not Applicable
	20d	Present results of all sensitivity analyses conducted to assess the robustness of the synthesized results.	Not Applicable
Reporting biases	21	Present assessments of risk of bias due to missing results (arising from reporting biases) for each synthesis assessed.	Not Applicable
Certainty of evidence	22	Present assessments of certainty (or confidence) in the body of evidence for each outcome assessed.	Not Applicable
**DISCUSSION**			
Discussion	23a	Provide a general interpretation of the results in the context of other evidence.	Pages 12-14 Lines 218-362
	23b	Discuss any limitations of the evidence included in the review.	Pages 14-15 Lines 364-371
	23c	Discuss any limitations of the review processes used.	Pages 14-15 Lines 364-371
	23d	Discuss implications of the results for practice, policy, and future research.	Page 15 Lines 382-386
**OTHER INFORMATION**			
Registration and protocol	24a	Provide registration information for the review, including register name and registration number, or state that the review was not registered.	Page 2 Lines 66-68

Section and Topic	Item #	Checklist item	Location where item is reported
	24b	Indicate where the review protocol can be accessed, or state that a protocol was not prepared.	Pages 2-3 Lines 62-133
	24c	Describe and explain any amendments to information provided at registration or in the protocol.	Pages 2-3 Lines 69-72, 117-133
Support	25	Describe sources of financial or non-financial support for the review, and the role of the funders or sponsors in the review.	Page 15 Line 392
Competing interests	26	Declare any competing interests of review authors.	Page 15 Line 397
Availability of data, code and other materials	27	Report which of the following are publicly available and where they can be found: template data collection forms; data extracted from included studies; data used for all analyses; analytic code; any other materials used in the review.	Page 15 Lines 395-396

From:

54. Page, M.J.; McKenzie, J.E.; Bossuyt, P.M.; Boutron, I.; Hoffmann, T.C.; Mulrow, C.D.; Shamseer, L.; Tetzlaff, J.M.; Akl, E.A.; Brennan, S.E.; et al. The PRISMA 2020 statement: An updated guideline for reporting systematic reviews. *BMJ* **2021**, *372*, n71. https://doi.org/10.1136/bmj.n71.

For more information, visit: http://www.prisma-statement.org/

Supplementary Material S2

Table. List of Excluded Studies.

Authors	Title	Year of publication	Reason for exclusion
Figueroa-Silva et al. [55]	Is it just a psoriasiform dermatitis?	2017	No documentation of neuroendocrine differentiation of the tumor or one of its metastases was mentioned in the article.
Kazama et al. [56]	Small cell prostate cancer producing syndrome of inappropriate secretion of antidiuretic hormone; A case report	2018	No English copy of the article
Bost et al. [57]	Malignant tumors in autoimmune encephalitis with anti-NMDA receptor antibodies	2018	No documentation of neuroendocrine differentiation of the tumor or one of its metastases was mentioned in the article.
Hansen et al. [58]	Pembrolizumab for advanced prostate adenocarcinoma: findings of the KEYNOTE-028 study	2018	Excluded based on the title
Hu et al. [59]	Pure small-cell carcinoma of the prostate presenting with increasing prostate-specific antigen levels: A case report and review of the literature.	2018	No documentation of a paraneoplastic syndrome was mentioned in the article.
Sekii et al. [60]	Development of ectopic adrenocorticotropic hormone syndrome in a patient with prostate cancer during combined androgen blockade therapy	2018	No English copy of the article
Miretti et al. [61]	Prostate carcinoma and syndrome of inappropriate antidiuretic hormone secretion	2018	No English copy of the article
Bhangoo et al. [62]	Reversible intrahepatic cholestasis in metastatic prostate cancer: An uncommon paraneoplastic syndrome.	2018	No documentation of neuroendocrine differentiation of the tumor or one of its metastases was mentioned in the article.
Agrawal et al. [63]	Disseminated intravascular coagulation as an initial manifestation of metastatic prostate cancer emergently treated with docetaxel-based chemotherapy.	2019	No documentation of neuroendocrine differentiation of the tumor or one of its metastases was mentioned in the article.

Tu et al. [64]	Large Cell Neuroendocrine Carcinoma of the Prostate: A Systematic Review and Pooled Analysis.	2019	No documentation of a paraneoplastic syndrome was mentioned in the article.
Kondo et al. [65]	Recurrent ataxia and respiratory failure with probable paraneoplastic syndrome responsive to plasma exchange therapy.	2019	No English copy of the article
Layman et al. [66]	Metastatic prostate cancer presenting as tumour-induced osteomalacia.	2019	No documentation of neuroendocrine differentiation of the tumor or one of its metastases was mentioned in the article.
Romašovs et al. [67]	Stauffer's syndrome in patient with metastatic prostate cancer.	2019	No documentation of neuroendocrine differentiation of the tumor or one of its metastases was mentioned in the article.
Brock et al. [68]	Co-occurrence of multiple endocrine neoplasia type 4 and spinal neurofibromatosis: a case report.	2020	No documentation of neuroendocrine differentiation of the tumor or one of its metastases was mentioned in the article.
Winther-Larsen et al. [69]	Hyperfibrinolysis in Patients with Solid Malignant Neoplasms: A Systematic Review	2020	No documentation of neuroendocrine differentiation of the tumor or one of its metastases was mentioned in the article.
Ghafouri et al. [70]	Exfoliative dermatitis as a para-neoplastic syndrome of prostate adenocarcinoma: A rare case report with literature review.	2021	No documentation of neuroendocrine differentiation of the tumor or one of its metastases was mentioned in the article.
Figueiredo et al. [71]	Paraneoplastic giant cell arteritis and prostate cancer: A case report of a not common association.	2021	No documentation of neuroendocrine differentiation of the tumor or one of its metastases was mentioned in the article.
Lim et al. [72]	Disseminated intravascular coagulation following femoral nailing in a metastatic prostate carcinoma patient - A case report.	2021	No documentation of neuroendocrine differentiation of the tumor or one of its metastases was mentioned in the article.
Sehgal et al. [73]	Metastatic Carcinoma of Prostate as a Mimicker of SAPHO Syndrome.	2021	No documentation of neuroendocrine differentiation of the tumor or one of its metastases was mentioned in the article.
Miranda Baleiras et al. [74]	Paraneoplastic dermatomyositis and prostate cancer: Myopathy regression under cancer-directed therapy.	2021	No documentation of neuroendocrine differentiation of the tumor or one of its metastases was mentioned in the article.
Greenberg et al. [75]	Disseminated intravascular coagulation as the initial	2021	No documentation of neuroendocrine differentiation of the tumor or one of its metastases was mentioned in the article.

	presentation of metastatic prostate adenocarcinoma.		
Mitchell et al. [76]	Real-world use of bone-modifying agents in metastatic castration-sensitive prostate cancer.	2022	Excluded based on the title
He et al. [77]	Collective analysis of the expression and prognosis for LEM-domain proteins in prostate cancer.	2022	Excluded based on the title
Apiraksattayakul et al. [78]	AQP4-IgG-positive neuromyelitis optica spectrum disorder and temporally detected neoplasms: case report and systematic review.	2022	No documentation of neuroendocrine differentiation of the tumor or one of its metastases was mentioned in the article.
Constante et al. [79]	Dermatomyositis: A cancer red flag	2022	No documentation of neuroendocrine differentiation of the tumor or one of its metastases was mentioned in the article.
Alçada et al. [80]	Synchronous Double Primary Cancer Complicated With Severe Hypercalcemia.	2023	No documentation of neuroendocrine differentiation of the tumor or one of its metastases was mentioned in the article.
Jatoi et al. [81]	A Rare Case of Primary Hyperparathyroidism and Hypercalcemia of Malignancy Seen in a Patient With Prostate Adenocarcinoma.	2023	No documentation of neuroendocrine differentiation of the tumor or one of its metastases was mentioned in the article.
Santandreu-Morales et al. [82]	Non-obstructive jaundice as paraneoplastic syndrome of prostate carcinoma: Systematic review of published cases.	2023	no English copy of the article

References:

55. Figueroa-Silva, O.; Espasandín-Arias, M.; García-Martínez, F.J.; Fernández-Redondo, V.; Toribio, J. Is it just a psoriasiform dermatitis? Dermatol. Online J. 2017, 23, 18. https://doi.org/10.5070/d32311037272.
56. Kazama, A.; Saito, T.; Ishikawa, S.; Takeda, K.; Kobayashi, K.; Tanikawa, T.; Tomita, Y. Small cell prostate cancer producing syndrome of inappropriate secretion of antidiuretic hormone; A case report. Nihon Hinyokika Gakkai Zasshi 2018, 109, 233–236. https://doi.org/10.5980/jpnjurol.109.233.
57. Bost, C.; Chanson, E.; Picard, G.; Meyronet, D.; Mayeur, M.-E.; Ducray, F.; Rogemond, V.; Psimaras, D.; Antoine, J.-C.; Delattre, J.-Y.; et al. Malignant tumors in autoimmune encephalitis with anti-NMDA receptor antibodies. J. Neurol. 2018, 265, 2190–2200. https://doi.org/10.1007/s00415-018-8970-0.

58. Hansen, A.R.; Massard, C.; Ott, P.A.; Haas, N.B.; Lopez, J.S.; Ejadi, S.; Wallmark, J.M.; Keam, B.; Delord, J.-P.; Aggarwal, R.; et al. Pembrolizumab for advanced prostate adenocarcinoma: Findings of the KEYNOTE-028 study. Ann. Oncol. 2018, 29, 1807–1813. https://doi.org/10.1093/annonc/mdy232.
59. Hu, J.; He, T.; Jin, L.; Li, Y.; Zhao, Y.; Li, W.; Wei, B.; Mao, X.M.; Lai, Y.Q.; Ni, L.C. Pure small-cell carcinoma of the prostate presenting with increasing prostate-specific antigen levels: A case report and review of the literature. Mol. Clin. Oncol. 2018, 9, 197–200. https://doi.org/10.3892/mco.2018.1644.
60. Sekii, Y.; Yoshinaga, M.; Nakagawa, M.; Kishikawa, H.; Oka, K.; Nishimura, K. Development of ectopic adrenocorticotropic hormone syndrome in a patient with prostate cancer during combined androgen blockade therapy. Hinyokika Kiyo 2018, 64, 175–179. https://doi.org/10.14989/ActaUrolJap_64_4_175.
61. Miretti, V.S.; Ávila, R.A.; Sierra, J.T.; García, J.J.; Laborié, M.V. Prostate carcinoma and syndrome of inappropriate antidiuretic hormone secretion. Medicina (B Aires) 2018, 78, 290–293. Available online: https://www.ncbi.nlm.nih.gov/pubmed/30125258 (accessed on Day Month Year).
62. Bhangoo, M.S.; Cheng, B.; Botta, G.P.; Thorson, P.; Kosty, M.P. Reversible intrahepatic cholestasis in metastatic prostate cancer: An uncommon paraneoplastic syndrome. Mol. Clin. Oncol. 2018, 8, 609–612. https://doi.org/10.3892/mco.2018.1564.
63. Agrawal, K.; Agrawal, N.; Miles, L. Disseminated intravascular coagulation as an initial manifestation of metastatic prostate cancer emergently treated with docetaxel-based chemotherapy. Case Rep. Oncol. Med. 2019, 2019, 6092156. https://doi.org/10.1155/2019/6092156.
64. Tu, X.; Chang, T.; Nie, L.; Qiu, S.; Xu, H.; Huang, Y.; Bao, Y.; Liu, Z.; Yang, L.; Wei, Q. Large Cell Neuroendocrine Carcinoma of the Prostate: A Systematic Review and Pooled Analysis. Urol. Int. 2019, 103, 383–390. https://doi.org/10.1159/000499883.
65. Kondo, T.; Igari, R.; Sato, H.; Iseki, C.; Ishizawa, K.; Suzuki, K. Recurrent ataxia and respiratory failure with probable para-neoplastic syndrome responsive to plasma exchange therapy. Rinsho Shinkeigaku 2019, 59, 339–344. https://doi.org/10.5692/clinicalneurol.cn-001234.
66. Layman, A.A.K.; Joshi, S.; Shah, S. Metastatic prostate cancer presenting as tumour-induced osteomalacia. BMJ Case Rep. 2019, 12, e229434. https://doi.org/10.1136/bcr-2019-229434.
67. Romašovs, A.; Puķītis, A.; Mokricka, V.; Frolova, E. Stauffer's syndrome in patient with metastatic prostate cancer. Case Rep. Urol. 2019, 2019, 9745301. https://doi.org/10.1155/2019/9745301.
68. Brock, P.; Alvarez, J.B.; Mortazavi, A.; Roychowdhury, S.; Phay, J.; Khawaja, R.A.; Shah, M.H.; Konda, B. Co-occurrence of multiple endocrine neoplasia type 4 and spinal neurofibromatosis: A case report. Fam. Cancer 2020, 19, 189–192. https://doi.org/10.1007/s10689-019-00152-6.
69. Winther-Larsen, A.; Sandfeld-Paulsen, B.; Hvas, A.-M. Hyperfibrinolysis in Patients with Solid Malignant Neoplasms: A Sys-tematic Review. Semin. Thromb. Hemost. 2020, 47, 581–588. https://doi.org/10.1055/s-0040-1715795.
70. Ghafouri, S.R.; Escriba-Omar, A.; Wahdatyar, I.; Whited, N.; Hakim, M.N.; Gaur, S.; Dihowm, F. Exfoliative dermatitis as a para-neoplastic syndrome of prostate adenocarcinoma: A rare case report with literature review. Cancer Diagn. Progn. 2021, 1, 289 - 295. https://doi.org/10.21873/cdp.10038.
71. Figueiredo, C.; Amaral, M.; Rodrigues, É.; Silva, R.; Vilão, Ó. Paraneoplastic giant cell arteritis and prostate cancer: A case report of a not common association. Clin. Case Rep. 2021, 9, 1405–1407. https://doi.org/10.1002/ccr3.3785.

72. Lim, J.W.S.; Zhang, W.; Park, D.H.; Premchand, A.X.R. Disseminated intravascular coagulation following femoral nailing in a metastatic prostate carcinoma patient—A case report. Trauma Case Rep. 2021, 36, 100534. https://doi.org/10.1016/j.tcr.2021.100534.
73. Sehgal, R.; Virata, A.R.; Bansal, P.; Hart, M. Metastatic Carcinoma of Prostate as a Mimicker of SAPHO Syndrome. Clin. Med. Res. 2021, 19, 141–147. https://doi.org/10.3121/cmr.2021.1539.
74. Baleiras, M.M.; Maduro, L.; Vasques, C.; Ferreira, F.; Pinto, M.M.; Martins, A. Paraneoplastic dermatomyositis and prostate cancer: Myopathy regression under cancer-directed therapy. Dermatol. Rep. 2021, 13, 9262. https://doi.org/10.4081/dr.2021.9262.
75. Greenberg, D.J. Disseminated intravascular coagulation as the initial presentation of metastatic prostate adenocarcinoma. Cureus 2021, 13, e14845. https://doi.org/10.7759/cureus.14845.
76. Mitchell, A.P.; Meza, A.M.; Panageas, K.S.; Lipitz-Snyderman, A.; Bach, P.B.; Morris, M.J. Real-world use of bone-modifying agents in metastatic castration-sensitive prostate cancer. J. Natl. Cancer Inst. 2022, 114, 419–426. https://doi.org/10.1093/jnci/djab196.
77. He, T.; Zhang, Y.; Li, X.; Liu, C.; Zhu, G.; Yin, X.; Zhang, Z.; Zhao, K.; Wang, Z.; Zhao, P.; et al. Collective analysis of the expression and prognosis for LEM-domain proteins in prostate cancer. World J. Surg. Oncol. 2022, 20, 174. https://doi.org/10.1186/s12957-022-02640-z.
78. Apiraksattayakul, N.; Songwisit, S.; Owattanapanich, W.; Tisavipat, N.; Siritho, S.; Prayoonwiwat, N.; Rattanathamsakul, N.; Jitprapaikulsan, J. AQP4-IgG-positive neuromyelitis optica spectrum disorder and temporally detected neoplasms: Case report and systematic review. Mult. Scler. Relat. Disord. 2022, 68, 104212. https://doi.org/10.1016/j.msard.2022.104212.
79. Constante, M.; Barradas, A.R.; Esteves, A.L.; Pereira, S.; Silva, L. Dermatomyositis: A cancer red flag. Cureus 2022, 14, e32502. https://doi.org/10.7759/cureus.32502.
80. Alçada, M.; Gaspar, V.; Cunha, G.; Manata, J.P.; Roque, F. Synchronous Double Primary Cancer Complicated with Severe Hypercalcemia. Cureus 2023, 15, e44272. https://doi.org/10.7759/cureus.44272.
81. Jatoi, A.; Haider-Badenhorst, Y. A Rare Case of Primary Hyperparathyroidism and Hypercalcemia of Malignancy Seen in a Patient with Prostate Adenocarcinoma. Cureus 2023, 15, e43497. https://doi.org/10.7759/cureus.43497.
82. Santandreu-Morales, I.; Redondo-Cerezo, E.; Martín-Enguix, D. Non-obstructive jaundice as paraneoplastic syndrome of prostate carcinoma: Systematic review of published cases. Med. Clin. 2023, 160, 206–212. https://doi.org/10.1016/j.medcli.2022.11.001.
